# Incidence of and risk factors for severe neutropenia during treatment with the modified FOLFIRINOX therapy in patients with advanced pancreatic cancer

**DOI:** 10.1038/s41598-022-18669-9

**Published:** 2022-09-16

**Authors:** Ai Irisawa, Misaki Takeno, Kazuo Watanabe, Hideaki Takahashi, Shuichi Mitsunaga, Masafumi Ikeda

**Affiliations:** 1 Department of Pharmacy, National Cancer Center Hospital East, Kashiwa, Japan; 2 Department of Analytical Biochemistry, Meiji Pharmaceutical University, Tokyo, Japan; 3 Department of Hepatobiliary and Pancreatic Oncology, National Cancer Center Hospital East, 6-5-1 Kashiwanoha, Kashiwa, Chiba 277-8577 Japan

**Keywords:** Gastroenterology, Oncology

## Abstract

Although FOLFIRINOX (l-Leucovorin/5-FU/Irinotecan/Oxaliplatin) is established as one of the standard therapies for patients with metastatic pancreatic cancer, the modified FOLFIRINOX (mFOLFIRINOX) is often used in clinical practice to reduce the incidence of toxicities. Febrile neutropenia (FN) and severe neutropenia during FOLFIRINOX are especially frequently observed in Japanese patients. In this study, we evaluated the incidence of FN and severe neutropenia, and explored the risk factors for severe neutropenia in patients receiving treatment with mFOLFIRINOX. The data of patients who had received mFOLFIRINOX between December 2013 and December 2014 at the National Cancer Center Hospital East were reviewed retrospectively. We graded the neutropenia severity and defined ≥ Grade 3 neutropenia as severe neutropenia. Univariate and multivariate analysis were undertaken to evaluate the associations with risk of development of severe neutropenia. A total of 122 patients were enrolled in this study. Sixty two patients (51%) and 10 patients (8%) developed severe neutropenia and FN, respectively. Multivariate analysis identified a low baseline white blood cell count (odds ratio [OR], 14.50; 95% confidence interval (CI), 3.27–111.14; *p* = 0.002) and presence of heterozygosity for UGT1A1*28 or UGT1A1*6 polymorphism (OR, 2.84; 95% CI, 1.18–7.17; *p* = 0.023) as independent risk factors for severe neutropenia. The incidences of severe neutropenia and FN in patients receiving mFOLFIRINOX in our clinical practice were comparable to previous reports. The risk factors for severe neutropenia in patients receiving mFOLFIRINOX were a low baseline white blood cell count and presence of heterozygosity for UGT1A1*28 or UGT1A1*6 polymorphism.

## Introduction

In 2022, the American Cancer Society estimates that 62,210 patients were diagnosed as having pancreatic cancer and 49,830 deaths were caused by pancreatic cancer^1^. In Japan, pancreatic cancer is the fourth leading cause of cancer-related death and the number of patients who died of the disease was 36,356 in 2019^2^.

For the treatment of metastatic pancreatic cancer (MPC), treatment with gemcitabine (GEM) alone, which was associated with a response rate of approximately 10% and yielded a median survival period of 5.65 months, remained the standard first-line chemotherapy until 2011^3,4^. Then, in the ACCORD11 trial, a phase II/III study performed in 2011, FOLFIRINOX (l-Leucovorin/5-FU/Irinotecan/Oxaliplatin) was demonstrated to significantly improve the overall survival (OS), progression-free survival (PFS), and quality of life as compared to GEM alone in patients with MPC^5^. In Japan, a phase II study conducted in 2014 demonstrated the efficacy of FOLFIRINOX as first-line treatment for MPC, and the treatment was approved in that setting^6^. Thereafter, a phase III study (MPACT) demonstrated better efficacy of GEM administered in combination with nab-paclitaxel (GEM + nab-PTX) as compared to GEM monotherapy in chemo-naive MPC patients^7^. It is difficult to compare the efficacy of GEM + nab-PTX with that of FOLFIRINOX, because the patient characteristics of the enrolled patients in the studies were slightly different. Therefore, these two regimens are currently considered as the standard first-line treatments for MPC in Japan. The main adverse effects of FOLFIRINOX are hematological toxicities, gastrointestinal symptoms, including nausea, vomiting, and diarrhea, and peripheral neuropathy. In particular, febrile neutropenia (FN) and grade 3/4 neutropenia were observed more frequently in a Japanese phase II study of FOLFIRINOX (22.2% and 77.8%) as compared to the ACCORD11 trial (5.4% and 45.7%)^5,6^. Therefore, a modified regimen of FOLFIRINOX (mFOLFIRINOX), in which the irinotecan dose is reduced to 150 mg/m^2^ and bolus 5-FU is omitted, is often administered in clinical practice to reduce the incidence of these toxicities. In a Japanese phase II study conducted in Japan to evaluate the efficacy and safety of mFOLFIRINOX in chemotherapy naïve MPC patients, mFOLFIRINOX showed comparable efficacy to, but was safer than the original regimen^8^. The NCCN guideline lists patient-related risk factors for FN obtained from systematic reviews, but most of the studies included in the systematic review are trials of lymphoma and breast cancer^9^. In addition, although there is some literature on the relationship between specific factors such as *UGT1A1* polymorphism and severe neutropenia for mFOLFIRINOX^10^, few studies until now have focused on the risk factors for severe neutropenia in patients treated with mFOLFIRINOX.

## Methods

This retrospective study was conducted with the approval of the ethics committee of the National Cancer Center, and in accordance with the Declaration of Helsinki (Approved No. 2014-352). We applied the opt-out method to obtain consent on this study. Informed consent for the treatment was obtained in writing from all patients.

### Patients

The study subjects were patients with advanced pancreatic cancer who had received treatment with mFOLFIRINOX between December 2013, when FOLFIRINOX treatment was reimbursed in Japan, and December 2014, when GEM + nab-PTX treatment began to be reimbursed, at the National Cancer Center Hospital East. After treatment with GEM + nab-PTX began to be reimbursed, we administered GEM + nab-PTX to the majority of patients as first-line chemotherapy, and reserved mFOLFIRINOX only for younger patients with a good performance status. Considering this selection bias, patients with special conditions were excluded from this study. Patients who were homozygous for UGT1A1*28 or UGT1A1*6 polymorphism, or with double-variant heterozygosity for UGT1A1*28 and UGT1A1*6 polymorphisms were excluded from this study, because these patients have already been reported to be at a high risk of development of severe neutropenia and received irinotecan at a reduced dose^11–13^. And the patients who received higher dose than mFOLFIRINOX were excluded.

### Treatment

The mFOLFIRINOX regimen consisted of oxaliplatin 85 mg/m^2^, irinotecan 150 mg/m^2^, and l-leucovorin 200 mg/m^2^ on day 1, followed by continuous intravenous infusion of 5-FU 2400 mg/m^2^ for 46 h. The treatment was repeated every 2 weeks. The doses of each agent could be adjusted based on the severity of adverse events. The treatment was continued until obvious disease progression or appearance of unacceptable toxicity. Prior to the treatment, a 5-HT_3_ receptor antagonist and dexamethasone were routinely given, and a selective neurokinin 1 receptor antagonist antiemetic was given from day1-3 to prevent nausea and vomiting induced by mFOLFIRINOX.

### Assessments

The incidences of severe neutropenia and FN were determined from the electronic charts of our hospital. All adverse events, including neutropenia and FN, were graded in severity according to the Common Toxicity Criteria for Adverse Events (CTCAE version 4.0). The highest grade of toxicity during the study period was used for the analysis. The risk factors for severe neutropenia among the pretreatment patient characteristics were explored.

### Relative dose intensity

The dose intensity was calculated by dividing the total dose of the agent administered by the number of weeks of treatment. The relative dose intensity (RDI) was calculated as the ratio of the actual dose intensity to that of the original regimen. The original regimen consisted of oxaliplatin 85 mg/m^2^, irinotecan 180 mg/m^2^, continuous intravenous infusion of 5-FU 2400 mg/m^2^, and bolus injection of 5-FU 400 mg/m^2^.

### Statistical analysis

Univariate and multivariate analysis were undertaken to evaluate the associations of the pretreatment clinical data with the incidence of severe neutropenia, and the clinical data were compared between the following two groups: patients who developed severe neutropenia (≥ Grade 3) at any time during the chemotherapy and those who did not. The univariate associations between the incidence of severe neutropenia and the pretreatment clinical data were analyzed by the Chi-square test or Fisher’s exact test. Multivariate logistic regression analysis was undertaken to identify factors significantly associated with the incidence of severe neutropenia. We entered all the factors that were identified as being significant by univariate analysis with a significance level of less than 0.2 into the multivariate analysis model. All the statistical analyses were performed using R version 4.0.3 (R Core Team (2020). R: A language and environment for statistical computing. R Foundation for Statistical Computing, Vienna, Austria. URL https://www.R-project.org/). All reported p-values are two-sided and *p* < 0.05 was considered as being indicative of statistical significance.

## Results

A total of 136 patients received mFOLFIRINOX between December 2013 and December 2014 at the National Cancer Hospital East; of these, 7 patients (5.1%) were excluded from this study because of administrating original FOLFIRINOX regimen, also 7 patients (5.1%) were excluded because of homozygosity for UGT1A1*28 or UGT1A1*6 or double-variant heterozygosity for UGT1A1*28 and UGT1A1*6. The remaining 122 patients were enrolled in this study. The baseline characteristics of the study subjects are summarized in Table 1. The median age of the patients was 65 years (range, 32–78 years), and 76 patients (62%) were male. The Eastern Cooperative Oncology Group (ECOG) performance status (PS) was 0 in 81 patients (66%) and 1 in 41 patients (34%). Of the 122, 96 patients (79%) had no history of prior chemotherapy. All the patients who were enrolled in this study underwent genetic analysis for determination of the *UGT1A1* genotype; 72 (59%) patients had wild-type *UGT1A1*, 27 (22%) were heterozygous for UGT1A1*6, and 23 (19%) were heterozygous for UGT1A1*28. The median follow-up period was 513 days [range: 307–672 days] and the median number of treatment cycles were 8 cycles [range: 1–28 cycles]. None of the patients received granulocyte-colony stimulating factor (G-CSF), including pegfilgrastim, for primary prophylaxis.

**Table 1:** Baseline patient characteristics.

Characteristics	ALL patients (N = 122)	Patients with severe neutropenia (N = 62)	Patients without severe neutropenia (N = 60)	*P* value
Median age (range), years	65 (32–78)	66 (39–78)	64 (32–77)	0.09
**Sex**				
Male	76 (62)	35 (56)	41 (68)	0.20
Female	46 (37)	27 (44)	19 (32)	
**ECOG PS**				
0	81 (66)	42 (68)	39 (65)	0.85
1	41 (34)	20 (32)	21 (35)	
**Treatment line**				
1st line	96 (79)	50 (81)	46 (77)	0.81
2nd line	12 (10)	5 (8)	7 (12)	
3rd line	14 (12)	7 (11)	7 (12)	
**Stage**				
Locally advanced	26 (21)	13 (21)	13 (22)	0.65
Metastasis	77 (63)	41 (66)	36 (60)	
Postoperative recurrence	19 (16)	8 (13)	11 (18)	
**Primary tumor location**				
Head	44 (36)	24 (39)	20 (33)	0.88
Body-tail	56 (46)	28 (45)	28 (47)	
None	20 (16)	9 (15)	11 (18)	
Other	2 (2)	1 (2)	1 (2)	
**Metastatic organs**				
Liver	56 (46)	31 (50)	25 (42)	0.37
Lymph node	40 (33)	18 (29)	22 (37)	0.44
Lung	21 (17)	10 (16)	11 (18)	0.81
**Baseline CEA (ng/mL)**^**a**^				
< 5.6	61 (50)	30 (48)	31 (52)	0.86
≥ 5.6	61 (50)	32 (52)	29 (48)	
**Baseline CA19-9 (U/mL)**^**a**^				
< 391.1	61 (50)	34 (55)	27 (45)	0.37
≥ 391.1	61 (50)	28 (45)	33 (55)	
**Biliary stent**				
Present	23 (19)	14 (23)	9 (15)	0.36
Absent	99 (81)	48 (77)	51 (85)	
**Peritoneal dissemination**				
No	86 (70)	46 (74)	40 (67)	0.43
Yes	36 (30)	16 (26)	20 (33)	
**Previous history of radiotherapy**				
Present	4 (3)	2 (3)	2 (3)	1.00
Absent	118 (97)	60 (97)	58 (97)	
*UGT1A1*(*6/*28)				
Wild type/wild type	72 (59)	32 (52)	40 (67)	0.05
Wild type/heterozygous	23 (19)	17 (27)	6 (10)	
Heterozygous/wild type	27 (22)	13 (21)	14 (23)	
**Baseline WBC (/mm**^**3**^**)**^**b**^				
≥ 4500	98 (80)	40 (65)	58 (97)	< 0.01
< 4500	24 (20)	22 (35)	2 (3)	
**Baseline Neu (/mm**^**3**^**)**^**b**^				
≥ 2160	113 (93)	54 (87)	59 (98)	0.03
< 2160	9 (7)	8 (13)	1 (2)	
**Baseline Lym (/mm**^**3**^**)**^**b**^				
≥ 1125	82 (67)	42 (68)	40 (67)	1.00
< 1125	40 (33)	20 (32)	20 (33)	
**Baseline Hb (g/dL)**^**b**^				
≥ 13 (male),12 (female)	60 (49)	26 (42)	34 (57)	0.15
< 13 (male),12 (female)	62 (51)	36 (58)	26 (43)	
**Baseline Plt (/mm**^**3**^**)**^**b**^				
≥ 120,000	112 (92)	55 (89)	57 (95)	0.32
< 120,000	10 (8)	7 (11)	3 (5)	
**Baseline CRP (mg/dL)**^**c**^				
≥ 0.3	65 (53)	36 (58)	29 (48)	0.36
< 0.3	57 (47)	26 (42)	31 (52)	
**Baseline AST (IU/L)**^**c**^				
< 40	102 (84)	54 (87)	48 (80)	0.33
≥ 40	20 (16)	8 (13)	12 (20)	
**Baseline ALT (IU/L)**^**c**^				
< 40	94 (77)	52 (84)	42 (70)	0.09
≥ 40	28 (23)	10 (16)	18 (30)	
**Baseline T-Bil (mg/dL)**^**c**^				
< 1.2	113 (93)	55 (89)	58 (97)	0.16
≥ 1.2	9 (7)	7 (11)	2 (3)	
**Baseline Alb (g/dL)**^**b**^				
≥ 3.8	79 (65)	35 (56)	44 (73)	0.07
< 3.8	41 (34)	25 (40)	16 (27)	
Missing	2 (2)	2 (3)	0 (0)	
**Baseline CCr (mL/min)**				
≥ 60	106 (87)	52 (84)	54 (90)	0.42
< 60	16 (13)	10 (16)	6 (10)	

The data are reported as the number (%) or median (range). The *p*-value was calculated by comparing patients with and without severe neutropenia.

*ECOG-PS* Eastern Cooperative Oncology Group-performance status, *WBC* white blood cell, *Neu* neutrophil count, *Lym* lymphocyte count, *Hb* hemoglobin level, *Plt* platelet count, *CRP* serum level of C-reactive protein, *AST* serum level of aspartate aminotransferase, *ALT* serum level of alanine aminotransferase, *T-Bil* serum level of total bilirubin, *Alb* serum level of albumin, *CCr* creatinine clearance.

^a^The median value was set as the cut-off value.

^b^The lower limit of normal was set as the cut-off value.

^c^The upper limit of normal was set as the cut-off value.

Of the 122 eligible patients, 62 patients (51%) and 10 patients (8%) developed severe neutropenia and FN, respectively (Table 2). Of the 62 patients who developed severe neutropenia, 40 patients (65%) developed grade 3 neutropenia, and 22 patients (35%) developed grade 4 neutropenia, with the time of onset in 43 patients (69%) in the first cycle, 8 patients (13%) in the second cycle and 11 patients (18%) in the third or later cycles. Of the 43 patients who developed severe neutropenia in the first cycle, 15 patients received G-CSF. Of the 10 patients who developed FN, the condition developed during the first cycle in 7 patients (70%), and during the second or later cycles in 3 patients (30%). Of the 7 patients who developed FN in the first cycle, 5 patients received G-CSF. The RDIs of oxaliplatin, irinotecan and continuous intravenous infusion of 5-FU were 68.3%, 66.7% and 77.8%, respectively (Table 3), and none of the patients received increased doses of the mFOLFIRINOX regimen.

**Table 2:** Incidence of severe neutropenia of current and previous study.

	mFOLFIRINOX, current study N = 122	mFOLFIRINOX, Japanese Phase II study^8^ N = 69	FOLFIRINOX, Japanese Phase II study^6^ N = 36
Grade 3–4 neutropenia, n (%)	62 (50.8)	33 (47.8)	28 (77.8)
Febrile neutropenia, n (%)	10 (8.2)	6 (8.7)	8 (22.2)

**Table 3:** Relative dose intensity.

	mFOLFIRINOX, current study N = 122 (%)	mFOLFIRINOX, Japanese Phase II study^8^ N = 69 (%)	FOLFIRINOX, Japanese Phase II study^6^ N = 36 (%)
Oxaliplatin	68.3	76.1	71.0
Irinotecan	66.7	91.4^a^	69.6
5-FU, bolus	–	–	15.9
5-FU, continuous infusion	77.8	95.4	80.3

^a^Relative dose intensity of irinotecan, 150 mg/m^2^.

To identify the risk factors for severe neutropenia, the associations between the incidence of grade 3/4 neutropenia and the patient characteristics were analyzed by univariate analysis (Table 4). The patients were classified into the “Non-grade 3/4 neutropenia” group (60 patients, 49%) and the “Grade 3/4 neutropenia” group (62 patients, 51%). The univariate analysis identified the following factors as being significant, with p-values of less than 0.2: female sex, heterozygosity for UGT1A1 UGT1A1*28 or UGT1A1*6, and a low baseline white blood cell (WBC) count, low baseline neutrophil count, low hemoglobin level, high serum alanine aminotransferase level, high serum total bilirubin level, and low serum albumin level at the study baseline. When these factors were entered into a multivariate analysis, only a low WBC count (odds ratio [OR], 14.50; 95% confidence interval (CI), 3.27–111.14; *p* = 0.002) and presence of heterozygosity for UGT1A1*28 or UGT1A1*6 polymorphism (OR, 2.84; 95% CI, 1.18–7.17; *p* = 0.023) were identified as independent risk factors (Table 4). Severe neutropenia was observed in 22 (91.7%) of the patients with low WBC count at the baseline, 30 (60%) of the patients with *UGT1A1* heterozygous for UGT1A1*28 or UGT1A1*6 polymorphism, and 10 (100.0%) of the patients had both of the above factors. The results of the risk factor analysis for severe neutropenia in patients who received mFOLFIRINOX as 1st line therapy are shown in Supplementary Table S1. These results were not different from the results in the overall enrolled patient population. In addition, Table 4 shows that the incidence of severe neutropenia was not significantly different between 1st line and 2nd or later line chemotherapies. Therefore, we conducted the analysis using the data of all patients.

**Table 4:** Risk factors for severe neutropenia among 122 patients treated with mFOLFIRINOX.

Variables	Number (%) of patients with severe neutropenia	Univariate analysis		Multivariate analysis	
		OR (95% CI)	*p*-value	OR (95% CI)	*p*-value
**Age (years)**					
< 65	25 (40.3)	1.57 (0.73–3.45)	0.209		
≥ 65	37 (59.7)				
**Sex**					
Male	35 (56.5)	1.65 (0.75–3.74)	0.176	1.46 (0.56–3.86)	0.436
Female	27 (43.5)				
**ECOG-PS**					
0	42 (67.7)	0.89 (0.39–2.01)	0.749		
1	20 (32.3)				
***UGT1A1* heterozygous**					
No	32 (51.6)	1.87 (0.85–4.17)	0.091	2.84 (1.18–7.17)	0.023
Yes	30 (48.4)				
**Peritoneal dissemination**					
No	46 (74.2)	0.70 (0.29–1.63)	0.362		
Yes	16 (25.8)				
**Biliary stent**					
Absent	48 (77.4)	1.65 (0.60–4.74)	0.285		
Preset	14 (22.6)				
**Liver metastasis**					
No	31 (50.0)	1.40 (0.64–3.05)	0.356		
Yes	31 (50.0)				
**Lymph node metastasis**					
No	44 (71.0)	0.71 (0.31–1.62)	0.369		
Yes	18 (29.0)				
**Stage**					
III	13 (21.0)	1.04 (0.40–2.72)	0.925		
IV	49 (79.0)				
**Baseline CEA (ng/mL)**^**a**^					
< 5.6	30 (48.4)	1.14 (0.53–2.46)	0.717		
≥ 5.6	32 (51.6)				
**Baseline CA19-9 (U/mL)**^**a**^					
< 391.1	34 (54.8)	0.68 (0.31–1.46)	0.277		
≥ 391.1	28 (45.2)				
**Treatment line**					
1st line	50 (80.6)	0.79 (0.30–2.06)	0.592		
2nd line or later	12 (19.4)				
**Previous history of radiation therapy**					
No	60 (96.8)	0.97 (0.07–13.75)	1.000		
Yes	2 (3.2)				
**Baseline WBC (/mm**^**3**^**)**^**b**^					
≥ 4500	40 (64.5)	15.64 (3.52–144.58)	< 0.001	14.50 (3.27–111.14)	0.002
< 4500	22 (35.5)				
**Baseline Neu (/mm**^**3**^**)**^**b**^					
≥ 2160	54 (87.1)	8.62 (1.10–393.53)	0.033	3.19 (0.22–84.74)	0.406
< 2160	8 (12.9)				
**Baseline Lym (/mm**^**3**^**)**^**b**^					
≥ 1125	42 (67.7)	0.95 (0.42–2.17)	0.899		
< 1125	20 (32.3)				
**Baseline Hb (g/dL)**^**b**^					
≥ 13 (male),12 (female)	26 (41.9)	1.80 (0.83–3.95)	0.104	1.54 (0.58–4.12)	0.386
< 13(male),12 (female)	36 (58.1)				
**Baseline Plt**^**b**^					
≥ 120,000	55 (88.7)	2.40 (0.51–15.12)	0.323		
< 120,000	7 (11.3)				
**Baseline CRP (mg/dL)**^**c**^					
≥ 0.3	26 (41.9)	1.48 (0.68–3.22)	0.282		
< 0.3	36 (58.1)				
**Baseline AST (IU/L)**^**c**^					
< 40	54 (87.1)	0.60 (0.19–1.74)	0.290		
≥ 40	8 (12.9)				
**Baseline ALT (IU/L)**^**c**^					
< 40	52 (83.9)	0.45 (0.17–1.16)	0.069	0.62 (0.22–1.72)	0.368
≥ 40	10 (16.1)				
**Baseline T-Bil (mg/dl)**^**c**^					
< 1.2	55 (88.7)	3.66 (0.66–37.57)	0.164	3.53 (0.68–27.15)	0.162
≥ 1.2	7 (11.3)				
**Baseline Alb (g/dL)**^**b**^					
≥ 3.8	35 (56.5)	1.95 (0.85–4.58)	0.083	2.56 (0.91–7.45)	0.077
< 3.8	25 (40.3)				
**Baseline CCr (mL/min)**					
≥ 60	52 (83.9)	1.72 (0.52–6.21)	0.316		
< 60	10 (16.1)				

*CI* confidence interval, *ECOG-PS* Eastern Cooperative Oncology Group-performance status, *WBC* white blood cell, *Neu* neutrophil count, *Lym* lymphocyte count, *Hb* hemoglobin level, *Plt* platelet count, *CRP* serum level of C-reactive protein, *AST* serum level of aspartate aminotransferase, *ALT* serum level of alanine aminotransferase, *T-Bil* serum level of total bilirubin, *Alb* serum level of albumin, *CCr* creatinine clearance.

^a^The median value was set as the cut-off value.

^b^The lower limit of normal was set as the cut-off value.

^c^The upper limit of normal was set as the cut-off value.

## Discussion

FN is associated with an increased risk of prolonged hospitalization, worse clinical outcomes, and life-threatening complications. Chemotherapeutic regimens that are associated with a high incidence of FN (> 20%) in chemo-naïve patients are considered as being high-risk regimens, and prophylactic administration of G-CSF is recommended in such patients^14^. Therefore, mFOLFIRINOX was devised to reduce the incidence of toxicities, including neutropenia and FN, associated with FOLFIRINOX. In this study, we retrospectively evaluated the incidence of FN and severe neutropenia associated with mFOLFIRINOX therapy, in order to identify the risk factors for severe neutropenia. The single-institution study was undertaken using the same unified method for tumor staging and identical treatment regimens and the same protocol for follow-up of blood sampling and administration of G-CSF, and we provided supportive care throughout, which enabled us to confirm important predictive factors.

The patient selection criteria for this study were broader than those of previous Japanese phase II studies, because this retrospective study was conducted in clinical practice. But, the patients homozygosity for UGT1A1*28 or UGT1A1*6 or double-variant heterozygosity for UGT1A1*28 and UGT1A1*6 were excluded from this analysis, because these patients treated with reduced dose of FOLFIRINOX in clinical practice. As compared to the Japanese phase II study of FOLFIRINOX, the median age of the enrolled patients was higher (65.0 years vs. 61.5 years) and the treatment line was not limited to 1st line treatment in this study. Nonetheless, the incidence of severe neutropenia and FN were lower than those reported for FOLFIRINOX^6^, and the results were consistent with those in the Japanese phase II study of mFOLFIRINOX^8^. The RDIs of oxaliplatin, irinotecan and continuous intravenous infusion of 5-FU in this study were comparable to those reported for FOLFIRINOX^6^ (Table 3). In addition, in 69% and 70% of cases, respectively, the severe neutropenia and FN occurred during the first cycle of treatment in this study. In the Japanese phase II study of FOLFIRINOX^6^, FN only occurred during the first cycle of treatment, so that the tendency seemed to be similar. Even in modified regimen, it is important to pay careful attention to severe neutropenia and FN especially during the first cycle and to undergo appropriate dose modification against the toxicities in the subsequent cycles.

In this study, we found that a low baseline WBC count and presence of heterozygosity for UGT1A1*28 or UGT1A1*6 polymorphism were significant independent risk factors for the development of severe neutropenia during treatment with mFOLFIRINOX. The NCCN guideline mentions that the most important risk factor for the development of severe neutropenia is advanced age, especially age > 65 years, in patients receiving chemotherapy at full dose intensity, and other risk factors include prior chemotherapy or radiotherapy, preexisting neutropenia or tumor invasion of the bone marrow, poor PS, comorbidities, including renal or liver dysfunction, HIV infection, and preexisting conditions such as neutropenia and infection^9^. In this study, the baseline WBC count was associated with the risk of severe neutropenia, this result was consistent with other chemotherapeutic regimens in previous studies^15–18^. On the other hand, we considered that low baseline neutrophil and low baseline WBC counts were cofounding factors, and low baseline neutrophil count was identified as one of the significant risk factors by univariate, but not by multivariate analysis.

*UGT1A1* is known to be involved in the metabolism of SN-38, an active metabolite of irinotecan, and double-variants of *UGT1A1* have been often reported to be risk factors for severe myelosuppression^12^. There are significant racial differences in the distribution of *UGT1A1* polymorphisms among Asians, Caucasians, and Africans. The frequency of UGT1A1*28 in Asians (16%) is one-third that in Caucasians (29–45%), and UGT1A1*6 is not detected at all in Caucasians or Africans, but is as frequent as the *28 allele in Asians (15–20%)^19^. Several studies have suggested that the incidence of severe neutropenia is significantly higher in patients with double-variant UGT1A1*28 and *6 heterozygosity than in those with the wild-type genotype. A meta-analysis suggested that the incidence of severe neutropenia is significantly higher in patients who heterozygous for UGT1A1*28 or *6 polymorphism than in patients with the wild-type genotype^20,21^. A previously reported prospective study on pancreatic cancer showed that the incidence of grade 3–4 hematological adverse events was higher in patients who were heterozygous for *UGT1A1* *6 or *UGT1A1* *28 than in patients with wild-type *UGT1A1*, although the difference did not reach statistical significance. However, the study suggested that the incidence of diarrhea was significantly higher in patients with heterozygous polymorphisms of UGT1A1*6 or *28 than in patients with the wild-type genotype^10^. Therefore, there appears to be convincing evidence to suggest that patients who are heterozygous for UGT1A1*6 or *28 polymorphism are at an increased risk for irinotecan toxicity as compared to patients with wild-type *UGT1A1*.

There were some limitations of this study. Firstly, the relatively small number of patients and there were only 50 patients who were heterozygous for UGT1A1*28 or UGT1A1*6 in the study made it difficult to draw any definitive conclusions. Furthermore, the study was a single-center and retrospective study, influenced by local individual clinician practices. Therefore, further clinical investigation, such as a multicenter trial is warranted to evaluate the risk factors for severe neutropenia associated with mFOLFIRINOX therapy in patients. Secondly, the safety data of mFOLFIRINOX were not evaluated in patients who were homozygous for UGT1A1*28 or UGT1A1*6 or heterozygous for both UGT1A1*6 and UGT1A1*28 in this study. However, a multicenter retrospective study of FOLFIRINOX in advanced pancreatic cancer patients with double-variant type UGT1A1*28 and *6 polymorphism was conducted by our colleagues and they recommend that the initial dose of irinotecan should be further reduced to ≤ 120 mg/m^2^ of body surface area in such patients^22^.

In conclusion, the incidences of severe neutropenia and FN were lower in the patients who received mFOLFIRINOX as compared to those reported for patients treated with FOLFIRINOX, despite the absence of significant differences in the relative dose intensities of the component drugs. The risk factors for severe neutropenia in patients receiving mFOLFIRINOX were a low baseline WBC count and heterozygosity for UGT1A1*28 or UGT1A1*6 polymorphism. Therefore, mFOLFIRINOX should be administered with caution in patients with these risk factors.

## Supplementary Information

Supplementary Table S1.

## Data Availability

The datasets analyzed during the current study are not publicly available because the ethical review and the informed consent for public release was not obtained, but are available from the corresponding author on reasonable request.
