# Supplementary material for: Incidence of and risk factors for severe neutropenia during treatment with the modified FOLFIRINOX therapy in patients with advanced pancreatic cancer
Source: Sci Rep. 2022 Sep 16;12:15574. doi: 10.1038/s41598-022-18669-9 (PMC9481868; doi:10.1038/s41598-022-18669-9)
Supplement: Supplementary file 1 — Supplementary Table S1. [file 41598_2022_18669_MOESM1_ESM.docx]

Incidence of and risk factors for severe neutropenia during treatment with the modified FOLFIRINOX therapy in patients with advanced pancreatic cancer

Supplementary Material

Ai Irisawa^1,2^, Misaki Takeno^1^, Kazuo Watanabe^3^, Hideaki Takahashi^3^, Shuichi Mitsunaga^3^, Masafumi Ikeda^3^

^1^ Department of Pharmacy, National Cancer Center Hospital East, Kashiwa, Japan

^2^ Department of Analytical Biochemistry, Meiji Pharmaceutical University, Tokyo, Japan

^3^Department of Hepatobiliary and Pancreatic Oncology, National Cancer Center Hospital East, Kashiwa, Japan

Correspondence to: Masafumi Ikeda, MD, PhD.

Department of Hepatobiliary & Pancreatic Oncology, National Cancer Center Hospital East, 6-5-1 Kashiwanoha, Kashiwa Chiba 277-8577, Japan

E-mail: masikeda@east.ncc.go.jp

Tel: +81-4-7133-1111

Fax: +81-4-7133-0335

**Supplementary Table S1. Risk factors for severe neutropenia among 96 patients treated with mFOLFIRINOX as first line treatment**

| **Variables** |  | **Number (%) of patients with severe neutropenia** | **Univariate analysis** | | **Multivariate analysis** | |
| --- | --- | --- | --- | --- | --- | --- |
|  |  |  | **OR (95% CI)** | ***p*-value** | **OR (95% CI)** | ***p*-value** |
| Age (years) | <65 | 21 (42.0) | 1.64 (0.68-3.99) | 0.226 |  |  |
|  | ≥65 | 29 (58.0) |  |  |  |  |
| Sex | Male | 29 (58.0) | 1.49 (0.60-3.76) | 0.342 |  |  |
|  | Female | 21 (42.0) |  |  |  |  |
| ECOG-PS | 0 | 36 (72.0) | 0.89 (0.34-2.36) | 0.793 |  |  |
|  | 1 | 14 (28.0) |  |  |  |  |
| *UGT1A1* heterozygous | No | 27 (54.0) | 1.93 (0.78-4.93) | 0.118 | 3.13 (1.08-9.83) | 0.041 |
|  | Yes | 23 (46.0) |  |  |  |  |
| Peritoneal dissemination | No | 38 (76.0) | 0.54 (0.20-1.42) | 0.167 | 0.26 (0.07-0.85) | 0.034 |
|  | Yes | 12 (24.0) |  |  |  |  |
| Biliary stent | Absent | 37 '74.0) | 2.32 (0.73-8.26) | 0.111 | 1.03 (0.20-5.34) | 0.974 |
|  | Preset | 13 (26.0) |  |  |  |  |
| Liver metastasis | No | 25 (50.0) | 1.55 (0.64-3.79) | 0.285 |  |  |
|  | Yes | 25 (50.0) |  |  |  |  |
| Lymph node metastasis | No | 34 (68.0) | 0.97 (0.38-2.51) | 0.949 |  |  |
|  | Yes | 16 (32.0) |  |  |  |  |
| Stage | III | 18 (36.0) | 1.04 (0.42-2.60) | 0.923 |  |  |
|  | IV | 32 (64.0) |  |  |  |  |
| Baseline CEA (ng/mL) ^a^ | <5.6 | 26 (52.0) | 1.10 (0.46-2.65) | 0.818 |  |  |
|  | ≥5.6 | 24 (48.0) |  |  |  |  |
| Baseline CA19-9 (U/mL) ^a^ | <391.1 | 25 (50.0) | 0.92 (0.38-2.20) | 0.831 |  |  |
|  | ≥391.1 | 25 (50.0) |  |  |  |  |
| Previous history of radiation therapy | No | 50 (100.0) | 0.00 (0.00-35.88) | 0.479 |  |  |
|  | Yes | 0 (0.0) |  |  |  |  |
| Baseline WBC (/mm^3^)^b^ | ≥4500 | 34 (68.0) | 20.66 (2.93-903.14) | <0.001 | 19.84 (2.56-532.78) | 0.017 |
|  | <4500 | 16 (32.0) |  |  |  |  |
| Baseline Neu (/mm^3^)^b^ | ≥2160 | 42 (84.0) | 8.42 (1.05-387.86) | 0.032 | 2.15 (0.07-62.12) | 0.613 |
|  | <2160 | 8 (16.0) |  |  |  |  |
| Baseline Lym (/mm^3^)^b^ | ≥1125 | 35 (70.0) | 1.21 (0.45-3.29) | 0.670 |  |  |
|  | <1125 | 15 (30.0) |  |  |  |  |
| Baseline Hb (g/dL)^b^ | ≥13 (male),12 (female) | 22 (44.0) | 2.60 (1.06-6.59) | 0.021 | 2.56 (0.75-9.25) | 0.139 |
|  | <13(male),12 (female) | 28 (56.0) |  |  |  |  |
| Baseline Plt^b^ | ≥120,000 | 45 (90.0) | 4.92 (0.52-241.34) | 0.206 |  |  |
|  | <120,000 | 5 (10.0) |  |  |  |  |
| Baseline CRP (mg/dL)^c^ | ≥0.3 | 20 (40.0) | 1.63 (0.68-3.98) | 0.231 |  |  |
|  | <0.3 | 30 (60.0) |  |  |  |  |
| Baseline AST (IU/L)^c^ | <40 | 42 (84.0) | 0.78 (0.24-2.56) | 0.648 |  |  |
|  | ≥40 | 8 (16.0) |  |  |  |  |
| Baseline ALT (IU/L)^c^ | <40 | 40 (80.0) | 0.47 (0.17-1.29) | 0.104 | 0.60 (0.17-1.96) | 0.406 |
|  | ≥40 | 10 (20.0) |  |  |  |  |
| Baseline T-Bil (mg/dl)^c^ | <1.2 | 43 (86.0) | 7.20 (0.87-336.78) | 0.061 | 7.49 (0.79-186.70) | 0.122 |
|  | ≥1.2 | 7 (14.0) |  |  |  |  |
| Baseline Alb (g/dL)^b^ | ≥3.8 | 28 (56.0) | 2.25 (0.86-6.15) | 0.067 | 2.67 (0.71-10.64) | 0.151 |
|  | <3.8 | 20 (40.0) |  |  |  |  |
| Baseline CCr (mL/min) | ≥60 | 42 (84.0) | 1.98 (0.49-9.72) | 0.280 |  |  |
|  | <60 | 8 (16.0) |  |  |  |  |

CI, confidence interval; ECOG-PS, Eastern Cooperative Oncology Group-performance status; WBC, white blood cell; Neu, neutrophil count; Lym, lymphocyte count; Hb, hemoglobin level; Plt, platelet count; CRP, serum level of C-reactive protein; AST, serum level of aspartate aminotransferase; ALT, serum level of alanine aminotransferase; T-Bil, serum level of total bilirubin; Alb, serum level of albumin; CCr, Creatinine clearance

^a^ The median value was set as the cut-off value.

^b^ The lower limit of normal was set as the cut-off value.

^c^ The upper limit of normal was set as the cut-off value.
